# Lifetime asthma incidence is related to age at onset and allergies in western Sweden

**DOI:** 10.1002/clt2.70015

**Published:** 2024-12-10

**Authors:** Reshed Abohalaka, Selin Ercan, Lauri Lehtimäki, Linda Ekerljung, Helena Backman, Fatma Zehra Uslu, Saliha Selin Ozuygur Ermis, Madeleine Rådinger, Bright I. Nwaru, Hannu Kankaanranta

**Affiliations:** ^1^ Department of Internal Medicine and Clinical Nutrition Krefting Research Centre Institute of Medicine Sahlgrenska Academy University of Gothenburg Gothenburg Sweden; ^2^ Allergy Centre Tampere University Hospital Tampere Finland; ^3^ Faculty of Medicine and Health Technology Tampere University Tampere Finland; ^4^ Department of Internal Medicine/Respiratory Medicine and Allergology The Sahlgrenska Academy University of Gothenburg Gothenburg Sweden; ^5^ Department of Public Health and Clinical Medicine Umeå University Umeå Sweden; ^6^ Department of Respiratory Medicine Seinäjoki Central Hospital Seinäjoki Finland

**Keywords:** allergy, asthma, incidence, late‐onset

## Abstract

Although asthma is more frequently diagnosed in childhood, a substantial proportion of cases manifests in adulthood. Nonetheless, few studies have comprehensively examined asthma incidence across different ages, genders, and asthma phenotypes. We conducted a retrospective evaluation of asthma incidence from birth to late adulthood, stratified by age, gender, and the presence or absence of allergies. Our analysis indicates that a significant number of asthma cases emerged in adulthood, particularly among middle‐aged women, with adult‐onset asthma surpassing childhood‐onset asthma after the age of 35 years. Additionally, allergic asthma was more common in younger than older individuals but decreases with age, ultimately leading to a higher proportion of non‐allergic asthma in older than younger individuals. These findings underscore the predominance of adult‐onset asthma among females and confirm the majority of allergic asthma in children, which declines with age. Additionally, increasing age is associated with increased incidence of non‐allergic asthma. Asthma heterogeneity should be considered in both clinical management and research.

To the editor,

Asthma is a heterogeneous disease, affecting between 2% and 10% of the population, with its prevalence rising in many countries.^1^ Various phenotypes of asthma, such as allergic, non‐allergic, and adult‐onset asthma, are frequently observed.^1^, ^2^ However, there is a lack of evidence regarding the incidence of asthma across different age groups, genders, and phenotypes.^3^, ^4^ The few studies that have investigated asthma incidence across a broad age range indicate that adult‐onset asthma may be quite common.^4^, ^5^, ^6^ These studies also reveal that asthma is more common in boys than in girls. Although male dominance diminishes during puberty, the precise age at which sex shift occurs remains unclear.^3^, ^7^ Furthermore, there is minimal data on the incidence of allergic and non‐allergic asthma over an extensive age span.^5^ This study retrospectively evaluated the incidence of asthma in both men and women, from birth to late adulthood, considering the presence or absence of allergies.

We utilized the West Sweden Asthma Study.^8^ Two surveys were performed in random samples of 30,000 and 50,000 individuals in 2008 and 2016, respectively. From the two surveys, a total of 42,621 responded to a self‐administered questionnaire that inquired about asthma, respiratory symptoms, allergies, environmental exposures and various risk factors (Figure 1A). A diagnosis of asthma by a physician was confirmed by a positive response to the question, *“Have you been diagnosed by a doctor as having asthma?”* The age at which asthma was diagnosed was subsequently determined through the follow‐up question, *“What age were you when asthma was diagnosed?”.* Individuals were classified as allergic if they responded affirmatively to the question, *“Do you have allergic eye or nose problems (hay fever) or any other allergic rhinitis?”* Asthma diagnosed at the age of 12 years or older was categorized as adult‐diagnosed asthma, whereas asthma diagnosed before the age of 12 was classified as childhood‐diagnosed asthma. The incidence of asthma diagnosis was assessed in 10‐year age groups using cross‐sectional data, based on previously established methodologies.^3^, ^7^ Individuals with missing data regarding asthma diagnosis, reporting an asthma diagnosis beyond their current age, or those affirming physician‐diagnosed asthma without providing the age of diagnosis, were excluded from the analysis. Comprehensive description of the methodology is provided in the supplement.

**FIGURE 1:**
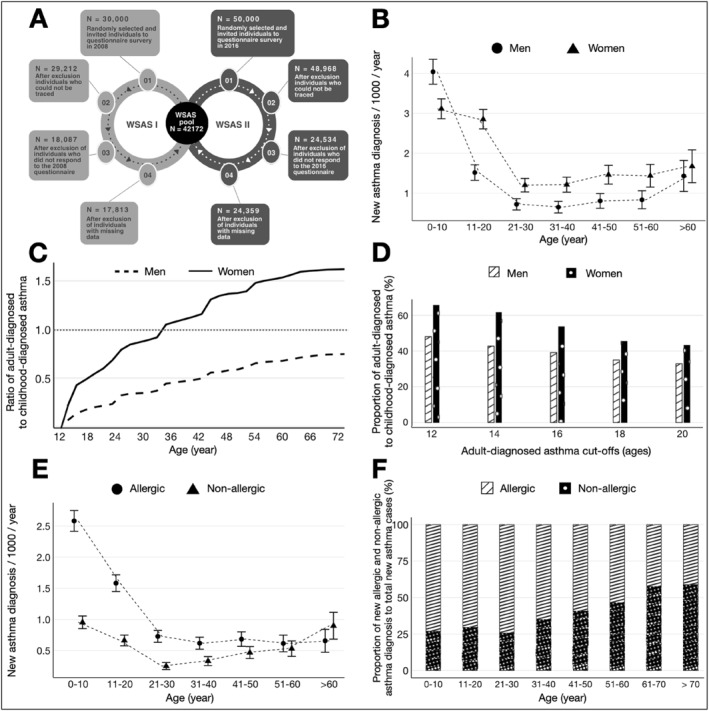
Asthma incidence while accounting for gender and allergy (*N* = 42,172). (A) Flow chart of the West Sweden Asthma Study, (B) asthma incidence stratified by gender, (C) The ratio of adult‐diagnosed asthma (≥12 years) to childhood‐diagnosed asthma (<12 years) stratified by gender, (D) the proportion of adult‐diagnosed asthma to childhood‐diagnosed asthma using different age cutoffs, (E) asthma incidence stratified by allergic conditions, (F) the proportion of new allergic and non‐allergic asthma diagnosis in relation to the total of new asthma cases in the age group.

Among all subjects, 3955 individuals (9.3%) reported having been diagnosed with asthma by a physician. Participants with asthma seemed to be younger, included a higher proportion of women, and exhibited more allergic conditions than those without asthma. Compared to survey participants in 2008, the prevalence of asthma was higher among those surveyed in 2016. However, there was a decrease in both current smoking rates and smoking history in those surveyed in 2016 than those surveyed in 2008 (Table 1). No other differences were observed between the two survey years.

**TABLE 1:** Characteristics of WSAS participants with and without asthma (*N* = 42,621).

	All participants		Participants surveyed in 2008		Participants surveyed in 2016	
	With asthma	Without asthma	With asthma	Without asthma	With asthma	Without asthma
*n* (%)	3955 (9.3)	38,666 (90.7)	1507 (8.3)	16,580 (91.7)	2448 (10.0)	22,086 (90.0)
Age (year)	45.7 (16.8)	48.3 (16.4)	44.0 (16.3)	46.2 (16.1)	46.9 (17.1)	50.0 (16.51)
Woman gender	2359 (59.7%)	20,868 (54.0%)	904 (60.0%)	8993 (54.2%)	1455 (59.4%)	11,875 (53.8%)
Age at asthma onset (year)	22.4 (18.5)	NA	22.0 (18.1)	NA	22.7 (18.7)	NA
Never smokers	2361 (59.7%)	23,606 (61.1%)	830 (55.1%)	9713 (58.6%)	1531 (62.5%)	13,893 (62.9%)
Current smokers	593 (15.0%)	5723 (14.8%)	282 (18.7%)	3086 (18.6%)	311 (12.7%)	2637 (11.9%)
Having allergic symptoms	2692 (68.1%)	10,553 (27.3%)	1034 (68.6%)	4377 (26.4%)	1658 (67.7%)	6176 (28.0%)

*Note*: Data was presented as *n* (%) or mean (SD). Allergic participants were those who responded affirmatively to the question, “*Do you have allergic eye or nose problems (hay fever) or any other allergic rhinitis*?”.

Abbreviations: NA, Non‐applicable; WSAS, West Sweden Asthma Study.

Asthma incidence was calculated in 10‐year age groups for both men and women (Figure 1B). Among men, the incidence peaked before the age of 10, followed by a low and stable incidence during adolescence and early adulthood, with a slight increase observed in later adulthood. In women, however, the incidence was lower during childhood and higher during adolescence and adulthood than in men. For the incidence of asthma in the separate survey years, please see the supplementary file.

Asthma diagnosed in childhood was more common than asthma diagnosed in adulthood in both men and women below the age of 35 years (Figure 1C). However, in women, the prevalence of adult‐diagnosed asthma surpassed that of childhood‐diagnosed asthma (i.e. became dominant) after the age of 35, while in men, childhood‐diagnosed asthma remained more common (Figure 1C). Furthermore, overall, adult‐diagnosed asthma was more common in women than in men. The higher prevalence in women than in men persisted even when the age cut‐off for adult‐diagnosed asthma was adjusted from 12 years to 14 and 16 years. However, when the cut‐off was adjusted to 18 or 20 years, childhood‐diagnosed asthma became more common in women as well (Figure 1D).

Upon calculating the incidence of asthma separately among allergic and non‐allergic participants, the incidence of asthma was significantly higher in allergic than non‐allergic participants at younger ages. However, this disparity gradually decreased with age, eventually reversing by the age of 60, at which point the incidence of asthma became higher among non‐allergic than allergic participants (Figure 1E). This pattern is corroborated by the analysis of the proportion of new asthma diagnoses, which indicated a marked decrease in new asthma cases among allergic individuals with age, alongside a rise in new asthma diagnoses among non‐allergic participants as they aged (Figure 1F).

The main limitation of our study is that asthma incidence was based on self‐reports, which may have introduced recall bias due to long time periods for some cases. However, this method is confirmed to reliably report the age of asthma onset.^9^ Another limitation could be the moderate response rate of 53.3%.

To summarize, our study indicates that although the incidence of asthma is higher in children than in adults, adult‐onset asthma is a common phenotype, particularly among women, where it is more common than childhood‐onset asthma. Additionally, the diagnosis of asthma in non‐allergic individuals becomes more common with aging than allergic asthma. These findings support previously published results,^3^, ^4^, ^5^, ^7^ although the incidence of adult‐onset asthma and the age of its predominance among women in our study was somewhat lower than in previous studies. In conclusion, our study suggests differences of asthma incidence between gender and asthma phenotypes, during the life course.

## AUTHOR CONTRIBUTIONS

**Reshed Abohalaka**: Conceptualization; methodology; software; data curation; investigation; validation; formal analysis; visualization; writing ‐ review and editing; writing ‐ original draft. **Selin Ercan**: Conceptualization; methodology; data curation; formal analysis; writing ‐ review and editing. **Lauri Lehtimäki**: Conceptualization; methodology; data curation; investigation; formal analysis; writing ‐ review and editing. **Linda Ekerljung**: Conceptualization; methodology; data curation; writing ‐ review and editing. **Helena Backman**: Methodology; conceptualization; data curation; supervision; writing ‐ review and editing. **Fatma Zehra Uslu**: Methodology; data curation; writing ‐ review and editing. **Saliha Selin Ozuygur Ermis**: Methodology; data curation; formal analysis; writing ‐ review and editing. **Madeleine Radinger**: Conceptualization; methodology; data curation; formal analysis; investigation; supervision; writing ‐ review and editing. **Bright I. Nwaru**: Conceptualization; methodology; data curation; software; investigation; supervision; writing ‐ review and editing. **Hannu Kankaanranta**: Conceptualization; methodology; data curation; investigation; validation; formal analysis; supervision; funding acquisition; writing ‐ review and editing.

## CONFLICT OF INTEREST STATEMENT

RA, SE, LL, LE, HB, FZU, MR, and BN: have no conflict of interest to declare. SSOE reports conference attendance‐related costs from Thermo Fisher Scientific outside the current study. HK reports fees for lectures and/or consulting from AstraZeneca, Boehringer‐Ingelheim, Chiesi Pharma, Covis Pharma, GSK, MSD, Novartis, Orion Pharma, and Sanofi outside the current study.

## Supporting information

Supporting Information S1

## Data Availability

The data that support the findings of this study are available on request from the corresponding author. The data are not publicly available due to privacy or ethical restrictions.
