# Supplementary material for: Lifetime asthma incidence is related to age at onset and allergies in western Sweden
Source: Clin Transl Allergy. 2024 Dec 10;14(12):e70015. doi: 10.1002/clt2.70015 (PMC11632114; doi:10.1002/clt2.70015)
Supplement: Supplementary file 1 — Supporting Information S1 [file CLT2-14-e70015-s001.docx]

**Supplementary file
Methods**

***Study population:***

The population of the West Sweden Asthma Study (WSAS) has been comprehensively delineated in a previous publication ^1^. To summarize, WSAS encompasses an initial cross-sectional examination of individuals aged 16 to 75 years. The inaugural survey was conducted in 2008, wherein a cohort of 30,000 individuals, selected at random to mirror the age and gender distribution of the study region, were solicited for participation. Of the initially targeted survey respondents, excluding those deemed untraceable (n=782), 18,087 individuals consented to take part in the survey. Subsequently, a second survey was executed in 2016, extending invitations to a randomly selected group of 50,000 individuals within the same age range as those included in the 2008 survey, with no overlapping participants between the two surveys. Overall, 24,534 individuals among the 50,000 invitees elected to participate in the 2016 survey, resulting in a cohort totalling 43,621 individuals from both surveys **(Figure S1**). Approval for the study was granted by the regional ethics board in Gothenburg, Sweden.


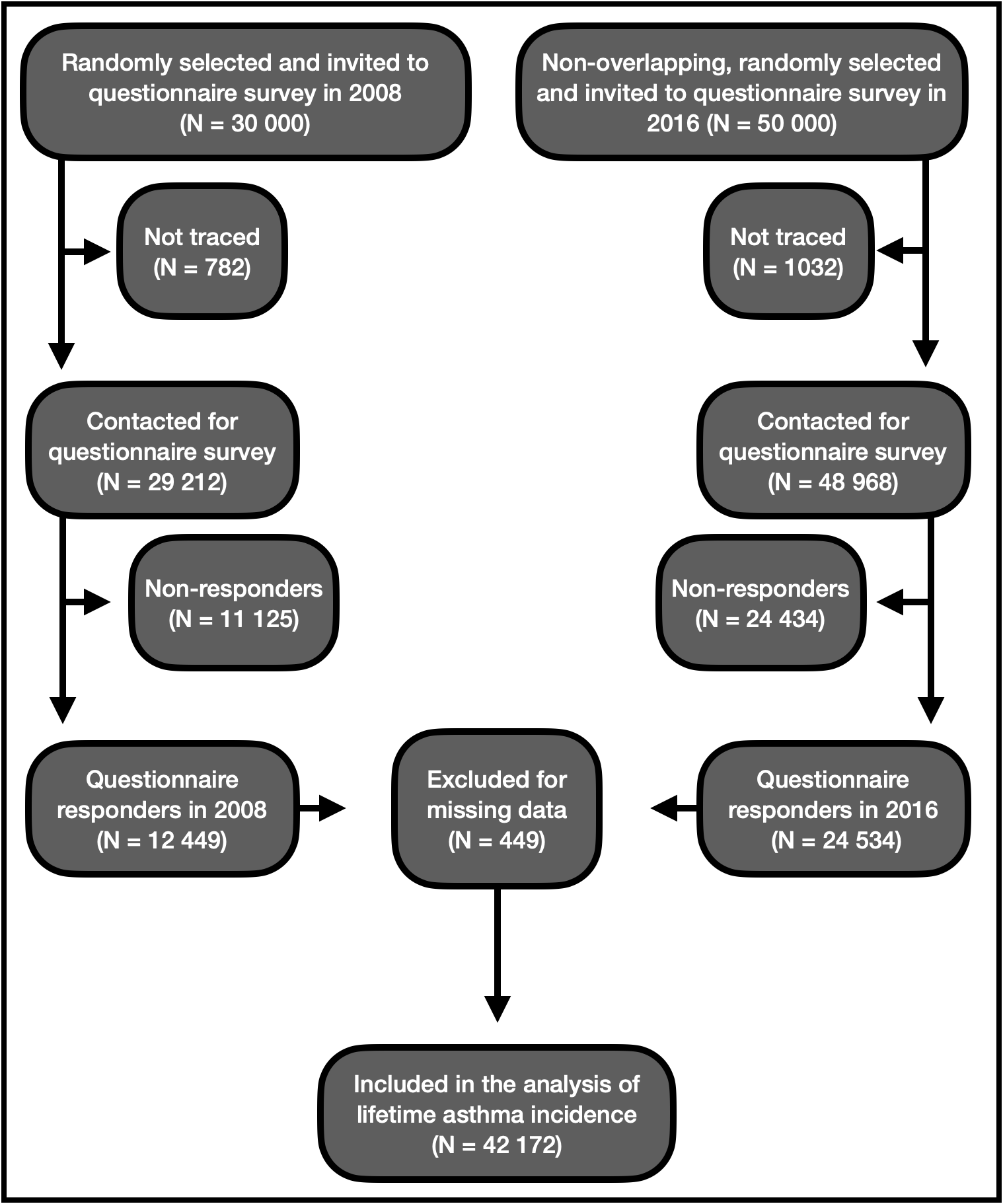


Figure S1: Flow chart of West Sweden Asthma Study (WSAS)

***Data collection and questionnaire***

At the outset of the study, participants were administered a postal self-administered questionnaire, which composed of inquiries previously employed in various epidemiological investigations including the Obstructive Lung Diseases in Northern Sweden (OLIN)^2,3^, the Global Allergy and Asthma European Network (GA_2_LEN) studies, the FinEsS studies conducted in Finland, Estonia, and Sweden^4,5^, and the European Community Respiratory Health Survey (ECRHS)^6^. Questions derived from the OLIN and ECRHS surveys primarily addressed topics related to asthma, rhinitis, chronic bronchitis, COPD, emphysema, respiratory symptoms, asthma medication usage, and potential risk factors encompassing smoking habits, family history of respiratory illnesses, occupational type, occupational and environmental exposures, comorbidities, and socioeconomic status. Furthermore, the GA_2_LEN questionnaire was integrated to incorporate additional detailed inquiries regarding rhinitis and eczema. Prior to implementation in the study, relevant sections of the questionnaire were translated into Swedish.

***Analysis of incidence of asthma***

Physician-diagnosed asthma was defined as a positive response to the inquiry*, "Have you been diagnosed by a doctor as having asthma?"* Subsequently, the age at which asthma was diagnosed was determined through a follow-up question, *"What age were you when asthma was diagnosed?"*. The incidence of asthma diagnosis was evaluated within 10-year age cohorts using cross-sectional data, consistent with previously described methodologies ^7,8^. To summarize, participants were stratified into 10-year age groups based on their present age, and the metric of "new asthma diagnoses per 1000 person-years" was computed by dividing the number of incident asthma diagnoses within each group by the population at risk specific to that age group, then dividing the result by 10, and finally multiplying by 1000. The population at risk for each 10-year age group was estimated as the mean value derived from annually calculated risks pertaining to that particular age range. All respondents were considered at risk from age 0 onwards. For example, individuals reporting asthma onset at 26 years of age contributed 10 person-years to the initial two age groups, and 6 years and 1 event to the 20- to 30-year age group. Conversely, individuals without a diagnosed asthma condition, aged 34 years, for instance, contributed a total of 10 person-years to the initial three age categories, while contributing 4 years with no events to the age group spanning from 30 to 40 years. Individuals reporting asthma diagnoses beyond their current age, or those affirming physician-diagnosed asthma but failing to provide the age of diagnosis (n = 429), were excluded from the analysis. However, the calculations of asthma incidence were corrected for these participants. Individuals classified as allergic were those who responded affirmatively to the question, *“Do you have allergic eye or nose problems (hay fever) or any other allergic rhinitis?”.*

***Statistical analysis***

Statistical analyses were conducted utilizing RStudio software (Version 2023.12.1 Build 402, Posit Software PBC). Continuous variables were assessed for their distribution, with normally distributed variables presented as mean (SD).


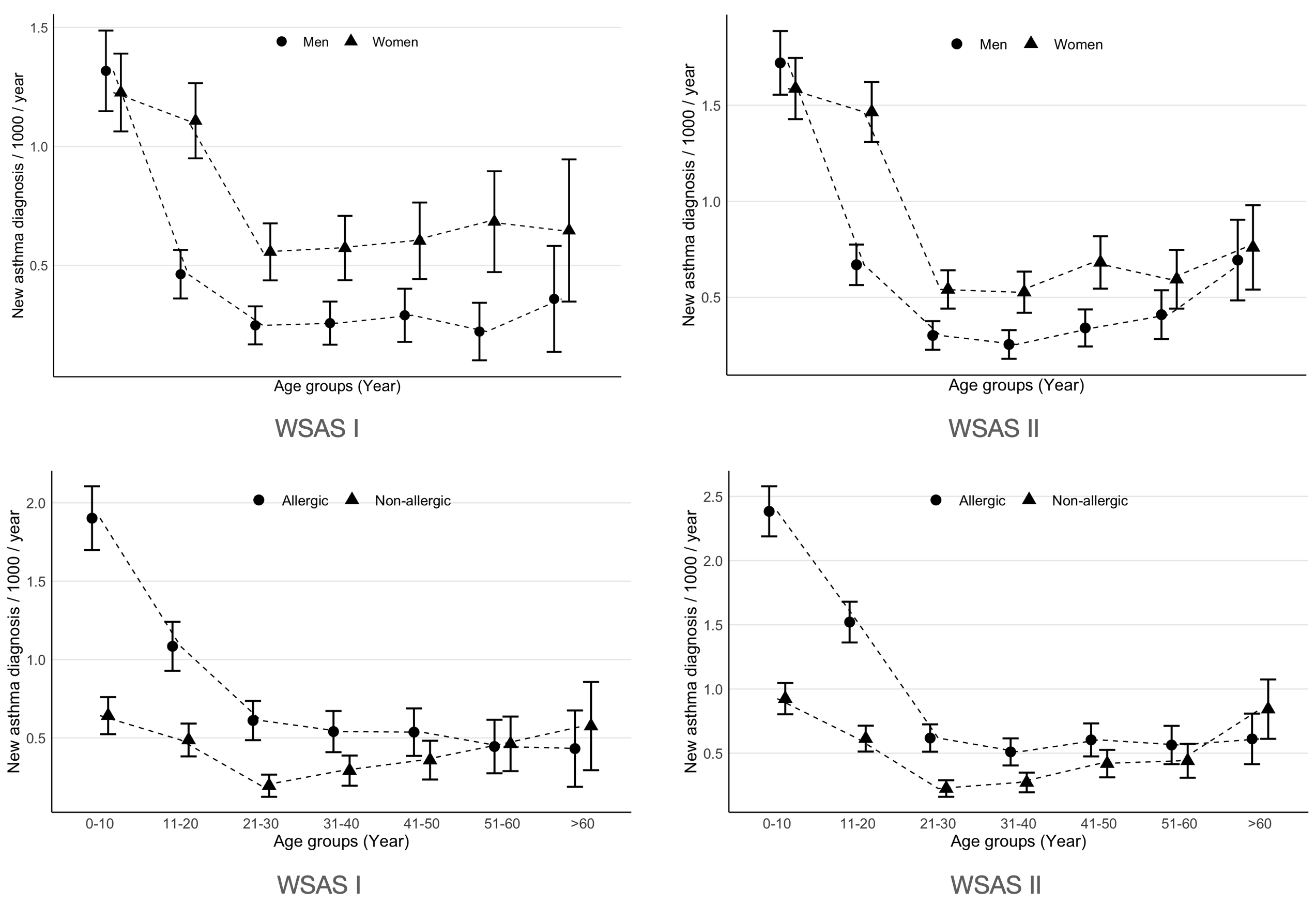


D

CA

B

A

**Figure S2**: Asthma incidence while accounting for gender (A, B) and allergy (C, D) calculated among WSAS I in 2008(A, C: n = 17,813) and WSAS II in 2016 (B, D: n = 24,359) participants separately.

References:

1. Nwaru BI, Ekerljung L, Rådinger M, et al. Cohort profile: the West Sweden Asthma Study (WSAS): a multidisciplinary population-based longitudinal study of asthma, allergy and respiratory conditions in adults. *BMJ Open*. Jun 19 2019;9(6):e027808. doi:10.1136/bmjopen-2018-027808

2. Rönmark E, Jönsson E, Lundbäck B. Remission of asthma in the middle aged and elderly: report from the Obstructive Lung Disease in Northern Sweden study. *Thorax*. Jul 1999;54(7):611-3. doi:10.1136/thx.54.7.611

3. Rönmark E, Lundbäck B, Jönsson E, Jonsson AC, Lindström M, Sandström T. Incidence of asthma in adults--report from the Obstructive Lung Disease in Northern Sweden Study. *Allergy*. Nov 1997;52(11):1071-8. doi:10.1111/j.1398-9995.1997.tb00178.x

4. Lindström M, Kotaniemi J, Jönsson E, Lundbäck B. Smoking, respiratory symptoms, and diseases : a comparative study between northern Sweden and northern Finland: report from the FinEsS study. *Chest*. Mar 2001;119(3):852-61. doi:10.1378/chest.119.3.852

5. Pallasaho P, Lindström M, Põlluste J, Loit HM, Sovijärvi A, Lundbäck B. Low socio-economic status is a risk factor for respiratory symptoms: a comparison between Finland, Sweden and Estonia. *Int J Tuberc Lung Dis*. Nov 2004;8(11):1292-300.

6. Burney PG, Luczynska C, Chinn S, Jarvis D. The European Community Respiratory Health Survey. *The European respiratory journal*. May 1994;7(5):954-60. doi:10.1183/09031936.94.07050954

7. Honkamäki J, Hisinger-Mölkänen H, Ilmarinen P, et al. Age- and gender-specific incidence of new asthma diagnosis from childhood to late adulthood. *Respir Med*. Jul-Aug 2019;154:56-62. doi:10.1016/j.rmed.2019.06.003

8. de Marco R, Locatelli F, Sunyer J, Burney P. Differences in incidence of reported asthma related to age in men and women. A retrospective analysis of the data of the European Respiratory Health Survey. *Am J Respir Crit Care Med*. Jul 2000;162(1):68-74. doi:10.1164/ajrccm.162.1.9907008
